# Dietary cadmium exposure assessment in rural areas of Southwest China

**DOI:** 10.1371/journal.pone.0201454

**Published:** 2018-08-02

**Authors:** Jiao Huo, Zhenzhen Huang, Renjia Li, Yang Song, Zhen Lan, Sijia Ma, Yongning Wu, Jinyao Chen, Lishi Zhang

**Affiliations:** 1 West China School of Public Health and Healthy Food Evaluation Research Center, Sichuan University, Chengdu, Sichuan, China; 2 Department of Nutrition and Food Safety, Sichuan Center for Disease Control and Prevention, Chengdu, Sichuan, China; 3 The Key Laboratory of Food Safety Risk Assessment, China National Center for Food Safety Risk Assessment, Beijing, China; University of Louisville School of Medicine, UNITED STATES

## Abstract

Dietary exposure of cadmium (Cd) has not been studied in Southwest China. The objective of the study was to determine the pollution characteristics and contamination levels in various agriculture products in Southwest China and to conduct a comparison of dietary exposure assessment of Cd in polluted and non-polluted areas. Results showed that the mean Cd contents in rice were 0.53 and 0.52 mg/kg in the high-polluted and low-polluted areas, respectively, with the average value was 0.03 mg/kg in the control area. The mean dietary Cd exposure from rice and vegetables of the selected non-occupational residents in Southwest China was 113.10 μg/kg bodyweight (bw)/month, 88.80 μg/kg bw/month, and 16.50 μg/kg bw/month in the high-polluted, low-polluted, and control areas, respectively, which correspond to 4.5 times, 3.6 times, and 0.66 times of the provisional tolerable monthly intake (25 μg/kg bw/month) established by the Joint FAO/WHO Expert Committee on Food Additives. The findings indicated that the risk for Cd exposure of residents was high due to home-grown food (most especially rice) being near polluted areas and is of great concern.

## Background and introduction

Human exposure to heavy metals is one of the most significant risk factors and has been the subject of numerous scientific studies, with cadmium (hereafter Cd) being the most frequently investigated. Currently, the most convincing adverse effects of long-term Cd exposure are kidney injury [1], bone damage, and cancer [2]. The Joint FAO/WHO Expert Committee on Food Additives (JECFA) established a provisional tolerable monthly intake (PTMI) level of 25 μg/kg bodyweight (bw)/month [3].

Cd-contaminated food is the major source of Cd for the non-occupationally exposed population [4]. Cd is a toxicant with high rates of soil-to-plant transference. Edible plants grown on contaminated soils have been proved to be an important health concern due to the high content of Cd [5,6]. Cd levels in agricultural products vary widely, depending on plant varieties, soil types, and growing conditions [7,8]. Foods that are frequently consumed in large quantities, such as rice, wheat, cereal crops, and leafy vegetables, could be the most significant dietary sources of Cd [9–11]. The European Food Safety Authority recently reported that, across Europe, the average dietary intake of Cd in adults ranges between 7.6–12 μg/kg bw/month, and an intake of 10.0–15.6 μg/kg bw/month is considered high [11]. Both values are below the PTMI established by JECFA.

Among the top 10 countries for Cd refinery production, China was the first in 2014 [12]. In the past decades, environmental Cd pollution has worsened and, consequently, the contamination level of Cd in foods might increase. Rice is one of the most important staple foods in China; however, it is prone to take up Cd from soil. Considerable consumption of Cd-contaminated rice has been proved to be the main contributor of itai-itai disease, which occurred in Japan in the 1940s. Currently, no therapeutically effective metal chelating agents for reducing the Cd body burden exist; moreover, the cumulative dietary exposure of Cd is a public health concern [13]. Hence, the overall health risk needs to be explored and sub-populations at high risk from Cd exposure need to be identified.

China National Center for Food Safety Risk Assessment investigated Cd dietary exposure across the country with 228,687 food samples from 32 food categories. The results suggested that mean dietary Cd exposure of the general Chinese population (15.3 μg/kg bw/month) was below the PTMI of 25 μg/kg bw/month established by JECFA. Moreover, the report suggested that rice is the most important contributor to Cd exposure for the Chinese population, and people living in the southern areas of China are at higher risk than those in the northern areas [14]. Another study investigated Cd contamination in rice and vegetables and non-occupationally Cd exposure in South China and calculated the total monthly Cd exposure of subjects (70.98 mg/kg bw) in environmentally polluted areas, with rice contributing to 77% of total exposure [15]. Dietary Cd exposure assessment has been carried out in China (overall) [14], South China [5,15] and East China [16], however, it has not been reported in Southwest China, where rice serves as the main staple food. Cd pollution in Southwest China is uneven due to geographical and economic characteristics. Some areas are the major sources of pollution. Hence in this research, a detailed dietary exposure assessment of Cd was performed in specific environmentally polluted and non-polluted areas of Southwest China. The objective of the study was to determine the pollution characteristics and contamination levels in various agriculture products in Southwest China and conduct a comparison of dietary exposure assessment of Cd in the polluted and non-polluted areas.

## Material and methods

### Study area and preliminary investigation

The selected polluted area in Sichuan Province has been reported to be contaminated with Cd due to a history of mining and smelting. According to the results of the national food safety surveillance in Sichuan on Cd, home-grown rice products of 10 families of each administrative village of the three towns in the polluted and control areas were measured in the preliminary investigation (data not shown in detail). The areas with mean Cd concentrations >0.4 mg/kg in rice were chosen as the high-polluted, those with Cd concentrations between 0.2–0.4 mg/kg as the low-polluted, and those with Cd concentrations <0.05 mg/kg as the control. Economic conditions, geographic characteristics (rather than industry distribution), and lifestyle and dietary habits of the residents living in the control area were similar to those of the polluted areas, which were 200 km apart.

### Study population and dietary survey

Considering the cumulative toxicity and long half-life of Cd reported to be as long as 14 to 45 years [17,18], all residents aged 40–75 years old in the investigated areas fitting the inclusion criteria (residence over 15 years and mainly subsisted on locally grown rice and vegetables) were registered and randomly sampled in each age group (40–44, 45–49, 50–54, 55–59, 60–64, 65–69, and 70–75years). All subjects were randomly selected using stratified cluster sampling. Those exposed to occupational Cd (or other toxic metals, such as lead and mercury) were excluded, and residents with clinically diagnosed or clearly defined kidney problems, urological and reproductive diseases, severe hypertension, diabetes, and hyperparathyroidism were excluded.

Each participant in the present study was required to fill a health questionnaire (including demographics, present and previous places of residence, occupation, and health condition). A 24 h dietary recall survey for three days (including two weekdays and one weekend) was conducted to collect information, including meal locations, food categories, and raw materials and their estimated weight. All questionnaires used in the survey were adapted from normalized and validated ones that have been widely used in other studies recently [15,19]. The entire questionnaire was administered by trained interviewers. All participants gave written informed consent to the study, which was performed in accordance with the Declaration of Helsinki protocols and approved by the ethics committee of Sichuan University. More detailed information on questionnaire contents was available in supporting information S1 File and S2 File.

### Sample collection and analysis

Food samples were collected directly from the households of the survey participants, which included 100 rice and 100 vegetable samples from each area. In total, 300 rice samples, 119 stem vegetables, 88 leafy vegetables, and 93 root vegetables were collected. All samples were collected and stored in sealed polythene packages and immediately transported to the labs and frozen at −4°C until detection.

Only the edible parts of vegetable samples were used for analysis, and the concentrations of vegetables were determined on a fresh weight basis. A total of 1.00 g of samples was accurately weighted into polytetrafluoroethylene bottles, filled with 10 mL HNO_3_ (65%, Suprapur, Merck, Darmstadt, German) overnight, and then digested at 120°C for 8 h; 3 mL H_2_O_2_ (guaranteed reagent, Merck, Darmstadt, German) was added for another 2 h at 150°C, and then the samples were digested in 190°C and diluted with 10 mL purified water. The digested sample was diluted to a final volume of 50 mL by adding high-purity deionized water. Cd content was then determined by graphite electrode atomic absorption spectrometry following the protocol of the China National Food Safety Standard GB/T 5009.15–2014. All samples with results below the limit of detection (LOD) were assigned the value of LOD or half of it depending on the recommended method by WHO [20].

Two certificated reference materials (CRMs), GBW08510 (rice flour) and GBW10015 (Spinach), were purchased from National Research Center for Certified Reference Materials, China. Quality control procedures were conducted as follows: a blank solution and reference material samples were running each batch to check for contamination; a standard solution and reference material sample were analyzed in the process; and paralleled samples were set, and standard recovery test was conducted.

### Food consumption and exposure assessment

Food consumption of each participant was calculated according to their 3 day, 24 h dietary recall survey results. Point exposure assessment was conducted using models recommended by other studies [15,21]:

$$Exp_{i}=\sum_{j=1}^{n}\frac{F_{j}\times C_{j}}{W_{i}}\times 30$$

where *j* is the number of food categories, *F*_*j*_ is the individual consumption of food category (g/day), *C*_*j*_ is the median level of Cd contamination of the corresponding food category (mg/kg), and *W*_*i*_ is the average bw of the individuals (kg). A factor of 30 days was used to obtain a monthly exposure for each individual, which makes *Exp*_*i*_ comparable to the PTMI (25 μg/kg bw/month) recommended by JECFA.

### Statistical analysis

Using EpiData software, all information obtained were double input into a computer by two trained staff. All statistical calculations were performed using SPSS 17.0 for Windows. All parameters were reported as mean ± SD, median, and range values. One-way analysis of variance (ANOVA) was used after checking for homogeneity of variance and normality of data, or the Kruskal-Wallis rank sum test was performed. Comparisons between two groups were performed using t-test. *P*-values less than 0.05 were considered significant.

## Results and discussion

### Validation of analytical method

The accuracy of the analytical procedures was verified by analysis of appropriate certificated reference materials (CRMs) based on the same digestion and analytical methods. The measured values were 2.03 ± 0.04 mg/kg for rice flour CRM and 0.14 ± 0.03 mg/kg for spinach CRM, while the certificated values were (2.062 ± 0.052) mg/kg and (0.150 ± 0.025) mg/kg, respectively. Recoveries were ranged between 98.0–102.2% for rice flour CRM, and 97.3–101.4% for spinach CRM.

The LOD and the limit of quantification (LOQ) were calculated as three and ten times the standard deviation of ten measurements of a blank divided by the slope of the calibration curve in Cd spiked samples. The LODs ranged from 0.001 to 0.005 mg/kg, and the LOQs ranged from 0.003 to 0.017 mg/kg for different food groups.

### Study population characteristics

A total of 910 subjects (393 males and 517 females) were recruited with validated questionnaires (male to female ratio: 0.76:1). Average age of participants was 58.54±8.31 years. Distribution of age and gender of participants in each area is shown in Table 1.

**Table 1:** Demographic characteristics of the participants.

Age groups	High-polluted area (n = 436)		Low-polluted area (n = 225)		Control area (n = 249)	
	M	F	M	F	M	F
40–44	6	21	4	9	6	17
45–49	22	30	6	12	9	23
50–54	33	51	16	25	8	18
55–59	22	32	19	23	14	13
60–64	45	44	23	25	33	30
65–69	42	47	18	23	27	33
70–75	18	23	10	12	12	6
Total	188	248	96	129	109	140

### Cd contamination levels in food samples

A total of 300 rice samples, 119 stem vegetables (50 samples of garlic sprout, 37 samples of asparagus lettuce, and 32 samples of celery), 88 leafy vegetables (38 samples of edible rape and 50 samples of cabbage), and 93 root vegetables (40 samples of potato and 53 samples of radish) were collected. The results of Cd concentration in food samples were presented in Table 2 [22].

**Table 2:** Cd levels in major home-grown food samples from the polluted and control areas^a^.

Food group	Area	Sample number	Maximum (mg/kg)	Mean (mg/kg)	SD (mg/kg)	Median (mg/kg)	Detection rate^d^ (%)
Cereals							
Rice	High-polluted	153	1.73	0.53^b^	0.30	0.49	100.00
	Low-polluted	51	1.12	0.51^b^	0.22	0.52	100.00
	Control	96	0.46	0.03	0.01	0.01	88.54
Roots							
Radish	High-polluted	21	0.04	0.02	0.01	0.02	100.00
	Low-polluted	18	0.03	0.02	0.01	0.02	100.00
	Control	14	0.03	0.02	0.01	0.01	85.71
Stems							
Potato	High-polluted	10	0.05	0.03	0.01	0.03	100.00
	Low-polluted	13	0.04	0.02	0.01	0.02	100.00
	Control	17	0.06	0.02	0.01	0.01	70.59
Asparagus lettuce	High-polluted	11	0.13	0.08^b^^c^	0.04	0.07	100.00
	Low-polluted	12	0.05	0.03	0.01	0.02	100.00
	Control	14	0.04	0.03	0.01	0.03	100.00
Celery	High-polluted	9	0.36	0.19^b^^c^	0.12	0.14	100.00
	Low-polluted	10	0.15	0.08^b^	0.06	0.05	100.00
	Control	13	0.05	0.03	0.01	0.03	100.00
Leafy							
Garlic sprout	High-polluted	20	0.19	0.06^b^	0.04	0.05	100.00
	Low-polluted	15	0.12	0.04^b^	0.03	0.03	100.00
	Control	15	0.10	0.02	0.01	0.01	80.00
Edible rape	High-polluted	12	0.12	0.04	0.03	0.02	100.00
	Low-polluted	14	0.03	0.02	0.01	0.02	100.00
	Control	12	0.03	0.02	0.01	0.02	100.00
Cabbage	High-polluted	17	0.09	0.02	0.01	0.02	100.00
	Low-polluted	18	0.03	0.02	0.01	0.02	100.00
	Control	15	0.04	0.02	0.01	0.01	100.00

^a^ The vegetable concentrations were expressed on a fresh weight basis.

^b^ Significant difference of *p*<0.05 is observed compared with that in the control area.

^c^ Significant difference of *p*<0.05 is observed compared with that in the low-polluted area.

^d^ “Detection rate” was defined as the sample number of Cd concentrations above LODs per total number of samples.

The mean Cd concentration in rice in the control area was 0.03 mg/kg, which is similar to those in non-specific areas of China [14,23]. The detection rates of Cd in rice were 100% in the high-polluted and low-polluted areas, and the mean Cd concentrations in the polluted areas were higher than the Maximum Allowable Levels (MAL) in the food safety standards set by Chinese government (0.2 mg/kg) [22] or Codex Alimentarius Commission (CAC, 0.4 mg/kg) [24]. Due to diverse pollution characteristics of soils or water in study areas, mean Cd levels in polluted areas usually varied in different studies [15,25,26]. High concentrations of Cd in the studied regions might result from production processes of the smelt industries located nearby.

All mean levels of tested vegetable species from three study areas were lower than the MALs set by Chinese government (0.1–0.2 mg/kg fresh weight) [22] or CAC (0.05–0.2 mg/kg fresh weight) [24]. In high-polluted area, the mean levels in edible portions of vegetables (0.048 mg/kg fresh weight) were similar to the results from other contaminated areas in China [26,27]. Except for asparagus lettuce and celery in high-polluted area, the maximum Cd concentrations of all tested vegetable were below the MAL set by Chinese government. Considering the mean values, order of Cd concentration was celery > asparagus lettuce > garlic sprout > edible rape > potato > cabbage > radish. The Cd content in stem vegetables was the highest among the categories of vegetables. Furthermore, celery is suggested to be more capable of accumulating higher Cd levels than other vegetables in other studies [28,29], which is in accordance with the findings in this study. Our results showed that celery, even in the control area, accumulated the highest concentration of Cd among all categories of vegetables investigated. Besides, Cd levels in rice were higher than those in vegetables, probably due to differences in bioaccumulation efficiency. The measured range of rice Cd concentrations could vary from country to country by 3 orders of magnitude (0.01–1 mg/kg), depending on physiochemical properties of soils, cultivation practices and the genotype-environment interactions [30].

### Dietary Cd exposure assessment

The dietary pattern was similar between the polluted and control areas. Furthermore, the proportion of food consumption was nearly identical (data not shown). In this study, mean dietary Cd exposures from rice and vegetables of the selected non-occupational residents in Southwest China were 113.10, 88.80, and 16.50 μg/kg bw/month in the high-polluted, low-polluted, and control areas, respectively, which corresponded to 4.5 times, 3.6 times, and 0.66 times of the PTMI (25 μg/kg bw/month) established by JECFA. The significant difference of the Cd dietary exposure levels between the polluted and control areas were certainly attributed to the historical environmental contamination with smelt industries in the polluted areas. Average exposure levels in the two polluted areas were both higher than the average exposure levels of 70.98 μg/kg bw/month in adults aged over 40 years in a polluted area in South China [15]. The average exposure level in the control area was similar to the average adult exposure level of the southern Chinese population (19.5 μg/kg bw/month), as reported by Song et al. [14]. It’s suggested that steady Cd contents in human kidneys are expected to be achieved when a lifetime average exposure of 16.6 μg/kg bw/month was estimated for a person aged 50 years [3]. The average exposure level was close to the estimated exposure in the control area in this study.

**Fig 1:**
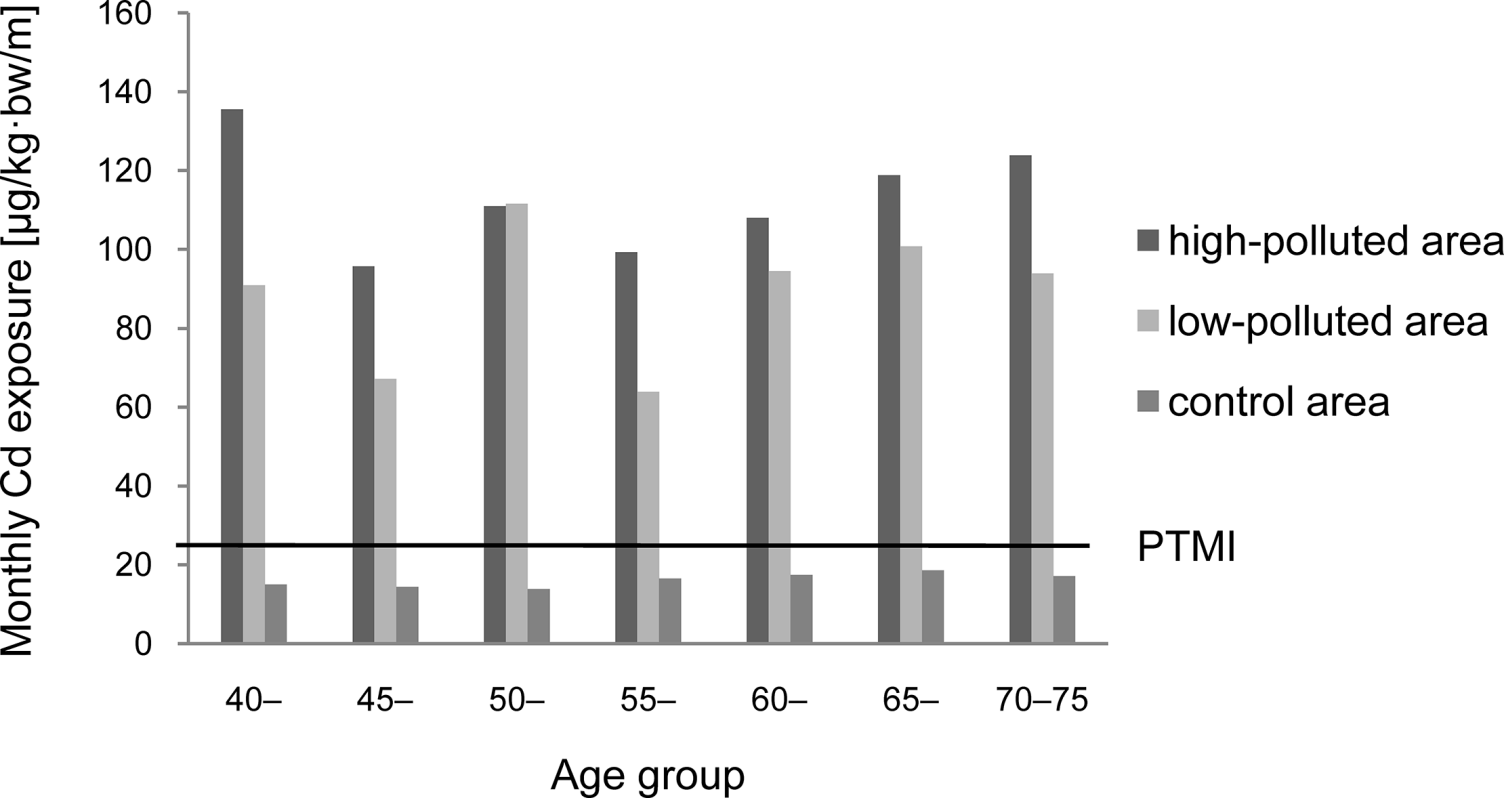
Monthly dietary Cd exposures of 40–75-year-old residents in the polluted and control areas in Southwest China.

Dietary Cd exposures from rice and vegetables of different age groups in the high-polluted, low-polluted, and control areas are shown in Table 3 and Fig 1. In the control area, the dietary Cd exposure remained relatively stable in all age groups (ranged from 13.80–18.60 μg/kg bw/month, 55%–74% of the PTMI). In the high-polluted area, Cd exposure of the 40–44 age group was the highest among all age groups (135.60 μg/kg bw/month, 5.42 times of the PTMI), while in the low-polluted area, those of the 50–54 age group was the highest (111.60 μg/kg bw/month, 4.46 times of the PTMI). The age difference of Cd exposure levels in polluted areas probably resulted from the variation in dietary intake among different age groups. However, the age trend was still unclear possibly due to the relatively small number of recruits in each subgroup.

**Table 3:** Dietary Cd exposures of the polluted and control areas.

Age group	Area	Monthly exposure (μg/kg bw/month)	Monthly exposure/PTMI^a^
40–44	High-polluted	135.60	5.42
	Low-polluted	90.90	3.64
	Control	15.00	0.60
45–49	High-polluted	95.70	3.83
	Low-polluted	67.20	2.69
	Control	14.40	0.58
50–54	High-polluted	111.00	4.44
	Low-polluted	111.60	4.46
	Control	13.80	0.55
55–59	High-polluted	99.30	3.97
	Low-polluted	63.90	2.56
	Control	16.50	0.66
60–64	High-polluted	108.00	4.32
	Low-polluted	94.50	3.78
	Control	17.40	0.70
65–69	High-polluted	118.80	4.75
	Low-polluted	100.80	4.03
	Control	18.60	0.74
70–75	High-polluted	123.90	4.96
	Low-polluted	93.90	3.76
	Control	17.10	0.68

^**a**^The ratio of the calculated Cd exposure to the PTMI of 25 μg/kg bw/month recommended by the JECFA.

### Contributions of rice and vegetables to Cd exposure

**Fig 2:**
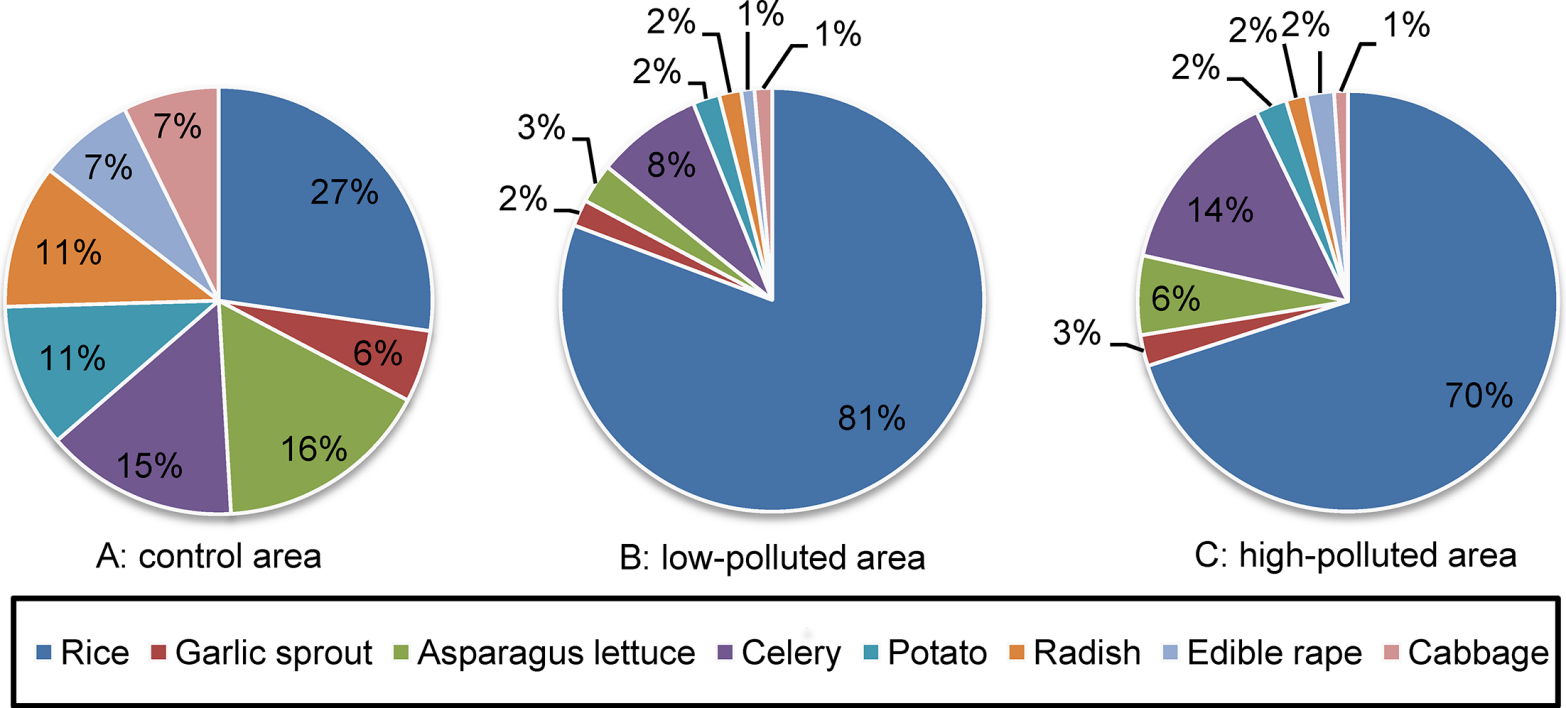
Contributions of different rice and vegetable groups to Cd exposure. (A) control area, (B) low-polluted area, (C) high-polluted area.

Cd exposures contributed by rice and different vegetables in the polluted and control areas are shown in Fig 2. Rice was the main contributor to total Cd dietary exposure in all three areas (27%, 81%, and 70% for the control, low-polluted, and high-polluted areas, respectively). In the control area, the second contributor was asparagus lettuce followed by celery, potato, and radish, all exceeding 10%. Moreover, celery, potato and asparagus lettuce are stem vegetables, radish is root vegetable. In the polluted areas, the Cd exposure contributions of all vegetable categories were similar, with celery being the highest, followed by asparagus lettuce. The contributions of other vegetable categories were relatively lower than or equal to 3%. In this study, the contribution of home-grown vegetables was 20%–30% in the polluted areas, which means the consumption of locally produced vegetables may contribute substantially to the total dietary Cd exposures.

For more than a decade, the food groups that contributed to Cd exposure have varied across countries. In the USA, the major contributors were seafoods (shellfish), wheat dishes and bread, and potatoes [31]. In Europe, grains and vegetables contributed the most [11]. Rice has been recognized as the most important contributor for northern (37.8%) and southern populations (65.1%) in China [14]. In this study, the contributions of rice to Cd exposure were over 70% in high- and low-polluted areas in Southwest China, presenting itself as a target for risk management. Cd intake through rice is also a potentially serious health risk for residents of Cd-contaminated areas in South China (about 70% of the total exposure) [15], and it should be of great concern for Cd risk reduction strategies. Rice is concluded to be the main contributor because it is a staple food and is grown on Cd-contaminated soil in Southwest and South China. In other Asian countries, rice has been reported to be the most important contributor of dietary Cd exposure, e.g., 90% in Vietnam [32] and 31% in Korea [33]. In addition, results of several studies performed in China showed that plant uptake factor of Cd in rice was higher than that in vegetables because of soil types [34,35], which suggested that more attention should be paid to the potential risk of Cd exposure form rice in China. The maximum permitted level for rice set by the CAC is at 0.4 mg/kg dry grain weight [24]; however, levels of 0.05–0.2 mg/kg are necessary to provide protection against Cd toxicity in those who consume an average of 300–400 g of cooked rice per day to meet physiological requirements. The Chinese government has set a MAL of 0.2 mg/kg of Cd in rice (lower than the CAC limit of 0.4 mg/kg) to protect public health [22].

Current studies pointed out that Cd in food is a widespread problem, and our results suggested that populations in the polluted area of Southwest China are exposed to high levels of Cd in their diet. Considering the possibility of multi-metal contamination in these areas, it is recommended that the actions should be taken to raise public awareness of self-management, in which attention should be paid to the source of food, vegetable species with high bioaccumulation capability, and the consumption frequency of food. Moreover, Cd-impacted soils need more special attention when trying to lower cadmium intakes by local government. Breeding low-Cd rice and improving cultivation practices could be two promising approaches for lowering Cd in local plants [30].

The main strengths of this study were that the investigation fills the gap of Cd exposure in China with the dietary assessment on the Cd exposure in both polluted and non-polluted areas in Southwest China, where rice serves as the main staple food, together with that our study provided details of Cd levels in different home-grown food samples in this area. However, we admitted that the present study has one limitation that the excluded criteria in our study included residents with different types of disease, some of which might have been related to a high intake of Cd, e.g., kidney and urological problems, thereby potentially leading to underestimation of the overall health risk. However, on account of the overall aim and tasks of the whole project which includes establishing a database of exposure biomarkers for heavy metals in China, we have to rule out residents with severe diseases under the project protocol so that the associations between biomarkers and cadmium exposure would be related. Future investigations based on the whole population will be conducted to better estimate the overall health risk of dietary exposure to Cd.

## Conclusion

To date, few studies have investigated dietary heavy metal exposure in populations in environmentally polluted areas. In this study, 910 subjects (40–75 years old) who subsisted with food cultivated from their lands were sampled. Health questionnaires and a 3 day, 24 h dietary recall survey was conducted for each individual. Moreover, food samples were collected to quantify Cd concentration and investigate Cd levels in rice and vegetables, and dietary Cd exposure assessment was performed in polluted and control areas in Southwest China.

Dietary Cd exposure from rice and vegetables in the polluted areas in Southwest China is of great concern. The residents who subsisted on home-grown food are at high risk. The dietary Cd exposure of the residents in the control area is relatively low for consumers who subsist on home-grown food, but the risk cannot be excluded. The health risk of Cd exposure of certain sub-groups is of great concern. An age difference was observed in dietary Cd exposure levels. Moreover, rice is the main contributor to dietary Cd exposure in the polluted and control areas. Stem vegetables should be considered when dealing with Cd contamination.

For the specific purpose of the study, only rice and vegetables were considered for the exposure assessment. Moreover, the overall exposure estimation would still be higher than the results presented. Furthermore, additional exposure pathways, such as smoking and inhalation, were not accounted for, which may play important roles for human Cd burden.

Contamination of soils with heavy metals, Cd in this case, is one of the biggest environmental problems due to agricultural plant accumulation. Thus, Cd exposure in the investigated population, particularly in the polluted areas in Southwest China, is a high-priority public health issue.

## Supporting information

S1 File Individual questionnaire. (DOC)

S2 File 24 hour dietary intake questionnaire. (DOCX)

S3 File Data sets. (XLSX)
