# Supplementary material for: Dietary cadmium exposure assessment in rural areas of Southwest China
Source: PLoS One. 2018 Aug 2;13(8):e0201454. doi: 10.1371/journal.pone.0201454 (PMC6072016; doi:10.1371/journal.pone.0201454)
Supplement: S1 File — (DOC) [file pone.0201454.s001.doc]

**Individual Questionnaire**

**Dietary Cadmium Exposure Assessment and Biological Monitoring**

| **Sample person ID:** | | **Place of interview:** | |
| --- | --- | --- | --- |
| **Interviewer ID: (signature)** | | **Reviewers ID: (signature)** | |
| **Date of interview:** | | **Date of review:** | |
| **Person answering questions is:** | **1 Subject** | | **2 Relative (specify: )** |
|  | **3 Acquaintance (specify: )** | | |

**SECTION 1 GENERAL QUESTIONS**

**PERSONAL INFORMATION**

| 1.1 | 1.1.1 | What is your full name: |  |
| --- | --- | --- | --- |
|  | 1.1.2 | Date of birth: □□□□/□□/□□(yyyy/mm/dd) 1 Lunar calendar 2 Solar calendar |  |
| 1.2 | National ID number：□□□□□□□□□□□□□□□□□ | |  |
| 1.3 | Gender: 1 Male 0 Female | |  |
| 1.4 | Place of primary residence: | | |
|  | 1.4.1 | How long do you live in the primary residence? □□/□□(yy/mm) | |
|  | 1.4.2 | Did you have homes elsewhere? 1 Yes 2 No | |
|  | 1.4.3 | If yes, please specify: where how long | |
| 1.5 | Do you eat local food? 1 Yes 2 No | | |
|  |  | If yes, did you eat any of the following food items? | |
|  | 1.5.1 | Rice 1 Yes 2 No if yes, please specify how many months did you eat during 1 year: early rice: □□ months per year late rice: □□ months per year | |
|  | 1.5.2 | Vegetables 1 Yes 2 No | |
|  | 1.5.3 | Beef, pork or lamb 1 Yes 2 No | |
|  | 1.5.4 | Poultry or eggs 1 Yes 2 No | |
|  | 1.5.5 | Fruits 1 Yes 2 No | |
|  | 1.5.6 | Water 1 Yes 2 No | |
|  | 1.5.7 | Cooking oil 1 Yes 2 No | |
|  | 1.5.8 | Other (specify): | |

**WOMEN ONLY**

| 1.6 | Did you live in the same place (town) before marriage? 1 Yes 2 No | |  |
| --- | --- | --- | --- |
|  | 1.6.1 | If no, please specify: place of residence before marriage:  marriage date: □□□□/□□(yyyy/mm) | |

**SECTION 2 occupational history**

| 2.1 | What is your current job? | |
| --- | --- | --- |
|  | 2.1.1 | How long did you stay at the current job? 1 <2 yeas 2 2~5years  3 5~10 years 4 10~15 years 5 >15 years |
|  | 2.1.2 | Does work involve industrial processes? 1 Yes 2 No  If yes, please specify: institution  describe work-related duties: |
| 2.2 | Did you have any other jobs previously? 1 Yes 2 No | |
|  | 2.2.1 | If yes, please specify: institution how long  describe work-related duties: |
| 2.3 | Does your current job involve any toxicants? 1 Yes 2 Doubt 3 Unknown 4 No | |
|  | 2.3.1 | If yes, please specify: |
|  | 2.3.2 | If doubt, please describe: |

**SECTION 3 HEALTH HABITS**

**Active smoking**

| 3.1 | Do you smoke tobacco? (smoke ≥1 cigarettes per day for at least one month)  1 Yes, presently go to question 3.2  2 Yes, in the past go to question 3.3  3 Not at all  4 Unknown | |
| --- | --- | --- |
| 3.2 | 3.2.1 | How old were you when you first started smoking: |
|  | 3.2.2 | Do you smoke tobacco every day? 1 Yes 2 No  If yes, how many years ago did you first begin smoking every day:  how many cigarettes do you smoke each day:  If no, how many cigarettes do you smoke per week: |
| 3.3 | 3.3.1 | How old were you when you first started smoking: |
|  | 3.3.2 | Did you smoke tobacco every day? 1 Yes 2 No  If yes, how many years ago did you first begin smoking every day:  how many cigarettes did you smoke each day:  If no, how many cigarettes did you smoke per week: |
|  | 3.3.3 | How long ago did you quit smoking: |

**Passive smoking**

| 3.2 | How often do you inhale secondhand tobacco smoke? (Inhalation time >15 min per day)  1 Every day 2 >3 days per week 3 1~3 days per week 4 Not at all 5 Unknown |
| --- | --- |

**SECTION 4 BACKGROUND** **MEDICAL** **HISTORY**

**PAST HISTORY**

| 4.1 | List any underlying diseases you have: (with the certificate of diagnosis provided by medical practitioner) | |
| --- | --- | --- |
|  | 4.1.1 | Anemia 1 Yes 2 No 3 Unknown  If yes, please specify: type: first diagnosed date□□□□ (yyyy) |
|  | 4.1.2 | Diabetes 1 Yes 2 No 3 Unknown  If yes, please specify: type: first diagnosed date□□□□ (yyyy) |
|  | 4.1.3 | Hypertension 1 Yes 2 No 3 Unknown  If yes, please specify: type: first diagnosed date□□□□ (yyyy) |
|  | 4.1.4 | Kidney disease 1 Yes 2 No 3 Unknown  If yes, please specify: type: first diagnosed date□□□□ (yyyy) |
|  | 4.1.5 | Liver disease 1 Yes 2 No 3 Unknown  If yes, please specify: type: first diagnosed date□□□□ (yyyy) |
|  | 4.1.6 | Thyroid disease 1 Yes 2 No 3 Unknown  If yes, please specify: type: first diagnosed date□□□□ (yyyy) |
|  | 4.1.7 | Heart disease 1 Yes 2 No 3 Unknown  If yes, please specify: type: first diagnosed date□□□□ (yyyy) |
|  | 4.1.8 | Seizures 1 Yes 2 No 3 Unknown  If yes, please specify: type: first diagnosed date□□□□ (yyyy) |
|  | 4.1.9 | Gout 1 Yes 2 No 3 Unknown  If yes, please specify: type: first diagnosed date□□□□ (yyyy) |
|  | 4.1.10 | Cancer 1 Yes 2 No 3 Unknown  If yes, please specify: type: first diagnosed date□□□□ (yyyy) |
|  | 4.1.11 | Reproductive system disease 1 Yes 2 No 3 Unknown  If yes, please specify: type: first diagnosed date□□□□ (yyyy) |
|  | 4.1.12 | Others 1 Yes 2 No 3 Unknown  If yes, please specify: first diagnosed date□□□□ (yyyy) |

**SYMPTOM REVIEW**

| 4.2 | Please check applicable symptoms and add additional as needed: | |
| --- | --- | --- |
|  | 4.2.1 | Have you had recent unexplained weight gain or loss? 1 Yes 2 No 3 Not sure  If yes, please specify: gain □ loss □ |
|  | 4.2.2 | Feeling hot or cold all the time 1 Yes 2 No 3 Not sure |
|  | 4.2.3 | Feeling sweaty or chills all the time 1 Yes 2 No 3 Not sure |
|  | 4.2.4 | Are you having trouble sleeping?  1 Yes 2 No 3 Not sure |
|  | 4.2.5 | Do you have:  Depression 1 Yes 2 No 3 Not sure  Irritability 1 Yes 2 No 3 Not sure  Difficulty concentrating 1 Yes 2 No 3 Not sure  Illusion 1 Yes 2 No 3 Not sure |
|  | 4.2.6 | Do you suffer with:  Headache or migraine 1 Yes 2 No 3 Not sure  Dizziness 1 Yes 2 No 3 Not sure  Ear or hearing problem 1 Yes, specify: 2 No 3 Not sure |
|  | 4.2.7 | Do you have:  Binge eating 1 Yes 2 No 3 Not sure  Binge drinking 1 Yes 2 No 3 Not sure |
|  | 4.2.8 | Do you have:  Pain or burning when urinating 1 Yes 2 No 3 Not sure  Blood in urine 1 Yes 2 No 3 Not sure  Change in urine color 1 Yes, specify: 2 No 3 Not sure  Frequent urination 1 Yes 2 No 3 Not sure  Strong, persistent urge to urinate 1 Yes 2 No 3 Not sure  Flank pain 1 Yes, specify: 2 No 3 Not sure |
|  | 4.2.9 | Do you have:  Joint or muscle pains 1 Yes, specify: 2 No 3 Not sure  Joint swelling, redness or deformity 1 Yes, specify: 2 No 3 Not sure  Osteoporosis 1 Yes 2 No 3 Not sure  Fracture 1 Yes 2 No 3 Not sure |
|  | 4.2.10 | Do you have tremors? 1 Yes, specify: 2 No 3 Not sure |
| 4.3 | For men | |
|  | 4.3.1 | Do you have:  Prostate problems 1 Yes 2 No 3 Not sure  Impaired libido  1 Yes 2 No 3 Not sure |
| 4.4 | For women | |
|  | 4.4.1 | At what age did your menstrual periods begin? □□  Number of pregnancies: □□  Number of live births: □□  Miscarriages: □□ |
|  | 4.4.2 | Are you still having menstrual periods?  1 Yes go to question 4.4.3  2 No go to question 4.4.4  3 Not sure |
|  | 4.4.3 | How often do they occur?  How many days do your periods last? □□(dd) |
|  | 4.4.4 | At what age did they stop? □□  Have you had any bleeding since menopause? 1 Yes 2 No 3 Not sure |
| 4.5 | Do you have any other information about your health that you would like us to known? | |

**Family Health History**

| 4.6 | Please check any of the diseases that run in your family and note who had it:  1 Anemia 2 Diabetes 3 Hypertension 4 Kidney disease 5 Liver disease  6 Thyroid disease 7 Heart disease 8 Seizures 9 Gout  10 Cancer 11 Reproductive system disease 12 Others | | | | |
| --- | --- | --- | --- | --- | --- |
|  | | | Age | Gender | Diseases type |
| Mother | | | □□ | M □ F □ | diseases □□ type: |
| Father | | | □□ | M □ F □ | diseases □□ type: |
| Children | | | □□ | M □ F □ | diseases □□ type: |
|  | | | □□ | M □ F □ | diseases □□ type: |
|  | | | □□ | M □ F □ | diseases □□ type: |
| Sibling | | Specify: | □□ | M □ F □ | diseases □□ type: |
|  | | Specify: | □□ | M □ F □ | diseases □□ type: |
|  | | Specify: | □□ | M □ F □ | diseases □□ type: |
