# Supplementary material for: Dietary cadmium exposure assessment in rural areas of Southwest China
Source: PLoS One. 2018 Aug 2;13(8):e0201454. doi: 10.1371/journal.pone.0201454 (PMC6072016; doi:10.1371/journal.pone.0201454)
Supplement: S2 File — (DOCX) [file pone.0201454.s002.docx]

**24-hour Dietary Intake Questionnaire
Individual Intake Form**

| **Sample Person Name:** | **Sample Person ID:** |
| --- | --- |
| **Interviewer ID: (signature)** | **Reviewers ID: (signature)** |
| **Date of Interview:** | **Date of Intake:** |

| **Time** | **Occasion** | **Food items** | **Description of food/drink and ingredient** | **How much of this food did you actually eat/drink?** | **Source of food** |
| --- | --- | --- | --- | --- | --- |
|  |  |  |  |  |  |
|  |  |  |  |  |  |
|  |  |  |  |  |  |
|  |  |  |  |  |  |
|  |  |  |  |  |  |
|  |  |  |  |  |  |
|  |  |  |  |  |  |
|  |  |  |  |  |  |
|  |  |  |  |  |  |
|  |  |  |  |  |  |
|  |  |  |  |  |  |
|  |  |  |  |  |  |
|  |  |  |  |  |  |
|  |  |  |  |  |  |
|  |  |  |  |  |  |
|  |  |  |  |  |  |
|  |  |  |  |  |  |
|  |  |  |  |  |  |
|  |  |  |  |  |  |
|  |  |  |  |  |  |
|  |  |  |  |  |  |
|  |  |  |  |  |  |
|  |  |  |  |  |  |
|  |  |  |  |  |  |
|  |  |  |  |  |  |

Occasion: 1. Breakfast 2. Lunch 3. Dinner 4. Late night meal

5. Snack 6. Other (specify):___________

Source of food: 1. Homemade 2. Restaurant 3. Street vender 4. Staff Canteen

5. Friend/Relative’s home 6. Other (specify): ___________
